# Dataset from fundus images for the study of diabetic retinopathy

**DOI:** 10.1016/j.dib.2021.107068

**Published:** 2021-04-21

**Authors:** Veronica Elisa Castillo Benítez, Ingrid Castro Matto, Julio César Mello Román, José Luis Vázquez Noguera, Miguel García-Torres, Jordan Ayala, Diego P. Pinto-Roa, Pedro E. Gardel-Sotomayor, Jacques Facon, Sebastian Alberto Grillo

**Affiliations:** a Department of Ophthalmology, Hospital de Clínicas, Facultad de Ciencias Médicas, Universidad Nacional de Asunción, San Lorenzo 2160, Paraguay; b Computer Engineer Department, Universidad Americana, Asunción 1029, Paraguay; c Facultad de Ciencias Exactas y Tecnológicas, Universidad Nacional de Concepción, Concepción 8700, Paraguay; d Division of Computer Science, Universidad Pablo de Olavide, ES-41013 Seville, Spain; e Facultad Politécnica, Universidad Nacional de Asunción, San Lorenzo 2160, Paraguay; f Department of Computer and Electronics, Universidade Federal do Espírito Santo, São Mateus, Brazil; g Universidad Autónoma de Asunción, Asunción, Paraguay

**Keywords:** Fundus images, Non-proliferative diabetic retinopathy, Proliferative diabetic retinopathy

## Abstract

This article presents a database containing 757 color fundus images acquired at the Department of Ophthalmology of the Hospital de Clínicas, Facultad de Ciencias Médicas (FCM), Universidad Nacional de Asunción (UNA), Paraguay. Firstly, the retinal images were acquired with a clinical procedure presented in this paper. The acquisition of the retinographies was made through the Visucam 500 camera of the Zeiss brand. Next, two expert ophthalmologists have classified the dataset. These data can help physicians and researchers in the detection of cases of Non-Proliferative Diabetic Retinopathy (NPDR) and Proliferative Diabetic Retinopathy (PDR), in their different stages. The dataset generated will be useful for ophthalmologists and researchers to work on automatic detection algorithms for Diabetic Retinopathy (DR).

## Specifications Table

**Table:** 

Subject	Ophthalmology.
Specific subject area	Diabetic Retinopathy.
Type of data	Image. Excel file.
How data were acquired	The fundus images were captured with the VISUCAM 500 camera from ZEISS [1]. The retinographies should be centered on the macula.
Data format	1. JPG (.jpg), Fundus images whose dimensions are 2124×2056.
	2. Annotations of the classifications in an EXCEL file (.xlsx).
Parameters for data collection	Field angle: 45${}^{\circ }$. Capture Mode: Color Image. Fixation points: Internal hexagonal fixation.
Description of data collection	Fundus images. We provide fundus images with Diabetic Retinopathy (DR) condition. The classification of fundus images have been done in 7 categories: No DR signs, Non-Proliferative Diabetic Retinopathy (NPDR) (mild, moderate, severe, very severe), Proliferative Diabetic Retinopathy (PDR) and Advanced PDR.
Data source location	The dataset of 757 color fundus images was acquired at the Department of Ophthalmology of the Hospital de Clínicas, Facultad de Ciencias Médicas, Universidad Nacional de Asunción, San Lorenzo 2160, Paraguay.
Data accessibility	Repository name: Dataset from fundus images for the study of DR Data identification number: http://doi.org/10.5281/zenodo.4647952 Direct URL to data: https://zenodo.org/record/4647952#.YGNjXVUzbIU

## Value of the Data

• The dataset is useful and important because it serves as a tool for both the medical scientific community and informatics researchers. Specifically, it helps to generate computational models for automatic diagnosis of the disease and for the training of new physicians in the staging of DR.
• Physicians can benefit from the dataset by using it in their professional training. Images with DR lesions assist in training physicians to analyze and detect the different stages of DR that can be seen on fundus images. At the same time, the dataset can help computer science researchers to build and validate new diagnostic tools for this disease.
• The dataset can be used to build and/or validate supervised and unsupervised learning models. The dataset is also useful for testing image processing algorithms.

## 1 Data Description

Retinal or fundus images are very useful in detecting multiple eye-related disorders. Retinal images can provide various types of structures such as blood vessels, the optic disc, the macula and the fovea. Information from these retinal image structures are used for the diagnosis and treatment of various retinal diseases [2], [3], [4].

According to the Early Treatment Diabetic Retinopathy Study (ETDRS), diabetic retinopathy can be classified as Non-Proliferative Diabetic Retinopathy (NPDR) and Proliferative Diabetic Retinopathy (PDR). NPRD is the early stage of the disease and is also subdivided into mild, moderate, severe, and very severe. Furthermore, PDR is the more advanced form of the disease and is additionally categorized into early and advanced. The medical experts classified the dataset into 7 categories considering the pathological conditions of DR. The dataset is separated into 7 folders corresponding to the seven categories of the disease (No DR signs, Mild (or early) NPDR, Moderate NPDR, Severe NPDR, Very Severe NPDR, PDR, and Advanced PDR). For more details on the classification criteria of fundus images, see [5]. The new dataset contains 757 fundus images of patients with diabetes, and the labels of each image and its diagnosis are provided in a file *Annotations of the classifications.xlsx*.

**Fig. 1:**
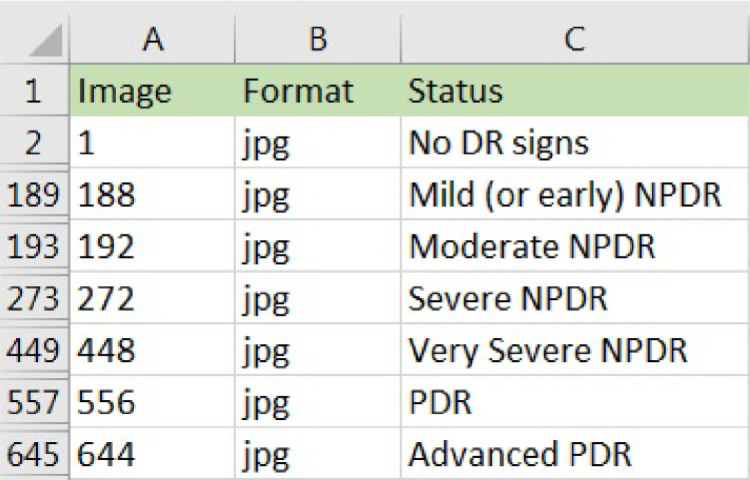
Example of DR labels in the XLSX file.

Fig. 1 shows information available in the XLSX file with the following column description:

A. The “Image” attribute is the number of the anonymized and renamed patient image.
B. The “Format” attribute shows the format of the image (.jpg).
C. The “Status” attribute shows the classification of the disease.

Table 1 shows the classification of the database and the number of fundus images owned by each category.

**Table 1:** Classification of the images in the database.

Classification	Number of images
No DR signs	187
Mild (or early) NPDR	4
Moderate NPDR	80
Severe NPDR	176
Very Severe NPDR	108
PDR	88
Advanced PDR	114

**Fig. 2:**
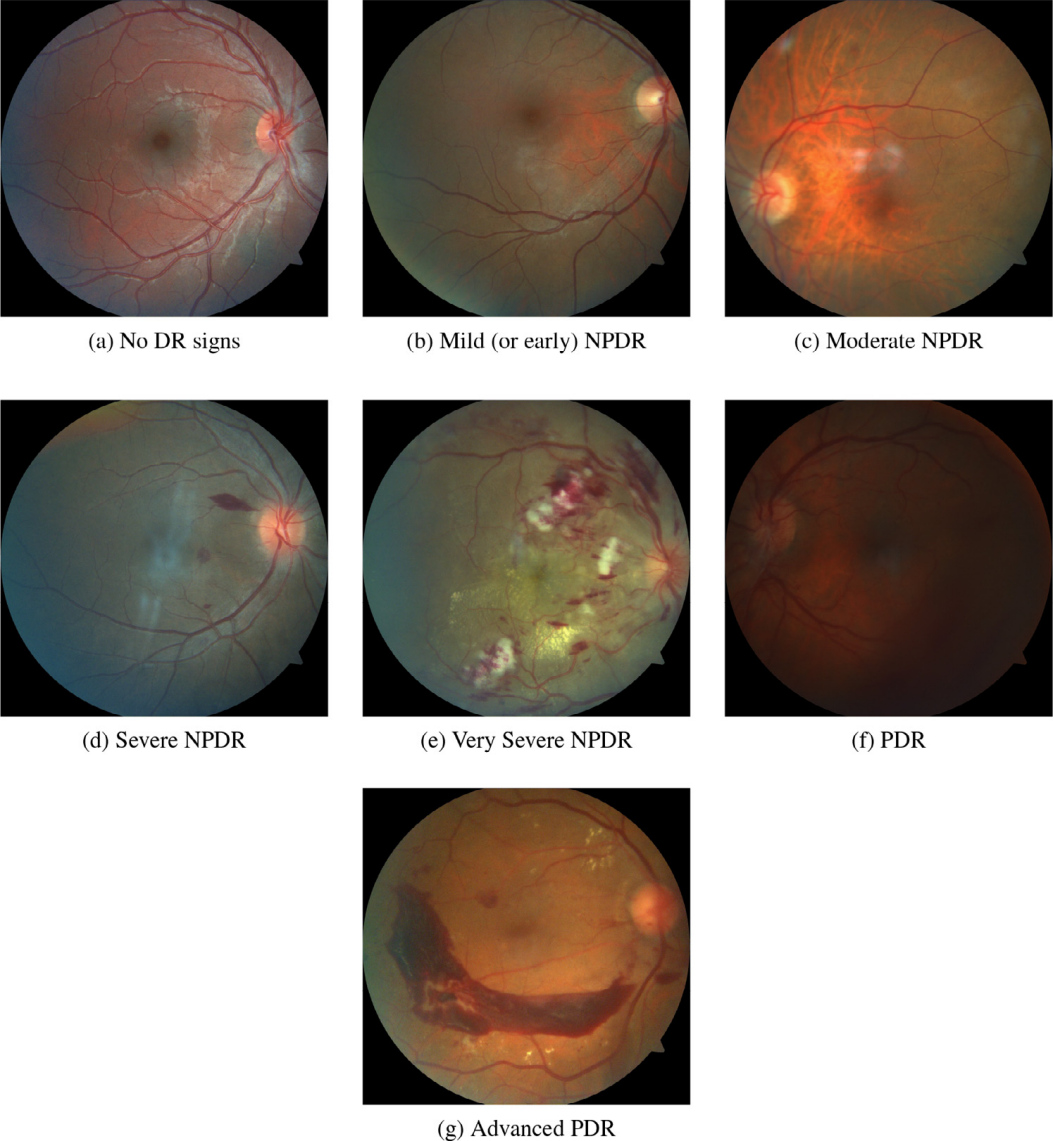
Examples of retinographies from the database.

Fig. 2 shows fundus images with different stages of DR.

## 2 Experimental Design, Materials and Methods

The acquisition of the 757 fundus images, from patients over 18 years old, was made following a procedure. These patients go to the fundus study service provided by the Department of Ophthalmology at the Hospital de Clínicas. The aforementioned procedure is described as follows:

1. Patients are interviewed and their data are recorded in a patient file.
2. Mydriatic eye drops are applied. A drop of *Fotorretin* (Phenylephrine 5% and Tropicamide 0,5% Laboratory Poen, Argentina) is applied every 15 minutes in each eye for 2 times. After 30 minutes and when pupil dilation is complete, the examination is performed in a dark room with the Visucam 500 camera [1]. The patient must be seated in front of the equipment and support the forehead and chin correctly.
3. A retinal image is captured from each eye using the Zeiss brand camera, model Visucam 500.
4. Next, a fundoscopic examination with an indirect ophthalmoscope and a 20D magnifier is performed. The staging of the diabetic retinopathy is established in the file of the patient. In the analysis of the obtained retinographies, the out-of-focus images, with artifacts or with low quality of the image due to media opacity (presence of corneal scars, cataracts or vitreous opacities such as hemovitreous or vitritis) are discarded. Retinographies of patients with other concomitant retinal pathology are not included.

## Ethics Statement

The use of the data in the research was authorized by the Department of Ophthalmology, Facultad de Ciencias Médicas, Universidad Nacional de Asunción. All patients signed an informed consent for treatment and examination. Their data remain anonymous, and their disease states are treated with the maximum confidentiality.

## CRediT Author Statement

**Veronica Elisa Castillo Benítez:** Conceptualization, Investigation, Resources, Data curation, Writing - original draft, Visualization; **Ingrid Castro Matto:** Conceptualization, Investigation, Re- sources, Data curation, Writing - original draft, Visualization; **Julio César Mello Román:** Inves- tigation, Resources, Data curation, Writing - original draft, Writing - review & editing, Visual- ization; **José Luis Vázquez Noguera:** Conceptualization, Validation, Resources, Writing - review & editing, Project administration, Funding acquisition; **Miguel García-Torres:** Conceptualization, Validation, Writing - review & editing, Project administration, Funding acquisition; **Jordan Ayala:** Data curation, Investigation, Visualization; **Diego P. Pinto-Roa:** Validation, Writing - review & editing; **Pedro E. Gardel-Sotomayor:** Validation, Writing - review & editing; **Jacques Facon:** Validation, Writing - review & editing; **Sebastian Alberto Grillo:** Validation, Writing - review & editing.

## Declaration of Competing Interest

The authors declare that they have no known competing financial interests or personal relationships that could have appeared to influence the work reported in this paper.

## Appendix A Supplementary materials

Supplementary Data S1 Supplementary Raw Research Data. This is open data under the CC BY license http://creativecommons.org/licenses/by/4.0/ Supplementary Data S1
