# Supplementary material for: Dataset from fundus images for the study of diabetic retinopathy
Source: Data Brief. 2021 Apr 21;36:107068. doi: 10.1016/j.dib.2021.107068 (PMC8257963; doi:10.1016/j.dib.2021.107068)
Supplement: Supplementary Data S1 — Supplementary Raw Research Data. This is open data under the CC BY license http://creativecommons.org/licenses/by/4.0/ [file mmc1.pdf]

UNIVERSIDAD NACIONAL DE ASUNCIÓN
FACULTAD DE CIENCIAS MÉDICAS
CÁTEDRA Y SERVICIO DE OFTALMOLOGÍA
Mariscal López e/ Cruzada de la Amistad – San Lorenzo
E-mail: oftalmologia@med.una.py
Tel: (0595 021) 683 930/2 Interno 298 y 115

San Lorenzo, 24 de marzo de 2021.-

Mrs.
Engr. Vanessa Cañete
INCADE S.A./ UNIVERSIDAD AMERICANA

I'am pleased to announce the authorization for the acquisition, processing, analysis and subsequent scientific publication of retinography images of our services, obtained with the **ZEISS VISUCAM 500** equipment belonging to the "Cátedra de Oftalmología del Hospital de Clínicas", within the framework of the project awarded by the CONACYT under the code: PINV18-846 Automatic detection of diabetic retinopathy using neuro-evolutionary algorithms.

Kind regards

Prof. Dr. Jorge E. Carrón
Oftalmólogo - Cirujano
Reg. Prof. 1567

Prof. Dr. Jorge E. Carrón, Jefe Interino
Cátedra de Oftalmología
Facultad de Ciencias Médicas
Universidad Nacional de Asunción
